# Role of ROS/RNS in Preeclampsia: Are Connexins the Missing Piece?

**DOI:** 10.3390/ijms21134698

**Published:** 2020-06-30

**Authors:** María F. Rozas-Villanueva, Paola Casanello, Mauricio A. Retamal

**Affiliations:** 1 Centro de Fisiología Celular e Integrativa, Facultad de Medicina Clínica Alemana, Universidad del Desarrollo, Santiago 7690000, Chile; fernanda.rozas.v@gmail.com; 2 Programa de Doctorado en Ciencias e Innovación en Medicina, Facultad de Medicina, Clínica Alemana Universidad del Desarrollo, Santiago 7690000, Chile; 3 Department of Obstetrics, School of Medicine, Pontificia Universidad Católica de Chile, Santiago 7690000, Chile; paolacasanello@gmail.com; 4 Department of Neonatology, School of Medicine, Pontificia Universidad Católica de Chile, Santiago 7690000, Chile; 5 Programa de Comunicación Celular de Cáncer, Facultad de Medicina Clínica Alemana, Universidad del Desarrollo, Santiago 7690000, Chile

**Keywords:** free radicals, connexins, hemichannels, preeclampsia, gap junction channels, vasculature

## Abstract

Preeclampsia is a pregnancy complication that appears after 20 weeks of gestation and is characterized by hypertension and proteinuria, affecting both mother and offspring. The cellular and molecular mechanisms that cause the development of preeclampsia are poorly understood. An important feature of preeclampsia is an increase in oxygen and nitrogen derived free radicals (reactive oxygen species/reactive nitrogen species (ROS/RNS), which seem to be central players setting the development and progression of preeclampsia. Cell-to-cell communication may be disrupted as well. Connexins (Cxs), a family of transmembrane proteins that form hemichannels and gap junction channels (GJCs), are essential in paracrine and autocrine cell communication, allowing the movement of signaling molecules between cells as well as between the cytoplasm and the extracellular media. GJCs and hemichannels are fundamental for communication between endothelial and smooth muscle cells and, therefore, in the control of vascular contraction and relaxation. In systemic vasculature, the activity of GJCs and hemichannels is modulated by ROS and RNS. Cxs participate in the development of the placenta and are expressed in placental vasculature. However, it is unknown whether Cxs are modulated by ROS/RNS in the placenta, or whether this potential modulation contributes to the pathogenesis of preeclampsia. Our review addresses the possible role of Cxs in preeclampsia, and the plausible modulation of Cxs-formed channels by ROS and RNS. We suggest these factors may contribute to the development of preeclampsia.

## 1. Introduction

Preeclampsia (PE) is a multisystemic disease of pregnancy, characterized by hypertension and proteinuria after 20 weeks of gestation [1]. Preeclampsia is associated with endothelial activation of the placental and maternal vasculature. The estimated prevalence of PE is 3–7% of pregnancies, which may vary according to the region, maternal age, ethnicity, and the socioeconomic status of the studied population [2]. Several risk factors for PE have been proposed, the most relevant being the presence of antiphospholipid antibodies, previous history of pre-eclampsia, and maternal diabetes [3,4]. PE is associated with several complications, such as hypertensive encephalopathy, eclampsia, renal and liver failure, pulmonary edema, coagulopathies, and uteroplacental dysfunction that may lead to intrauterine growth restriction [5]. Thus, PE is considered one of the leading causes of maternal and neonatal mortality and morbidity worldwide [6]. Furthermore, PE may exert its consequences weeks or years after pregnancy, in the form of cardiovascular risk and postpartum depression [7], chronic hypertension, metabolic syndrome, and cardiac structural changes leading to diastolic dysfunction [8]. Even though PE represents a major health problem, its etiology is not yet well understood. The main theory is based on a two-stage event: First, an inefficient invasion of the spiral arteries’ walls by the extravillous trophoblast during placentation, which leads to incomplete degradation of the muscle layer of the arterial wall, and therefore to high resistance and low flow of the placental vascular bed, generating a decreased uteroplacental perfusion. Second, the uteroplacental ischemia leads to hypoxia and necrotic damage of the trophoblast, which releases soluble factors such as fms-like tyrosine kinase 1 (sFLT1) and endoglin (sENG) and other inflammatory molecules, leading to endothelial dysfunction and its effects on maternal and fetal systemic disease [6,9,10]. However, further research is necessary to advance our understanding of the molecular mechanisms for PE and its sequels.

## 2. Inflammation and Redox Imbalance in Preeclampsia

Normal pregnancy has been identified as a transient inflammatory state because the site of implantation resembles an open wound. In this context, several groups have shown an improvement in in vitro fertilization success rates when a mechanical injury in the endometrium, like a biopsy, is done. It seems that the success of implantation may be dependent on an initial “injury-induced inflammatory reaction”, and in agreement with this idea, proinflammatory T helper-1 (Th)-1 cells and proinflammatory cytokines, such as IL-6, IL-8, and TNFα have been found in the implantation zone. However, this response has to be carefully regulated and is followed by an anti-inflammatory state, characterized by a shift towards Th-2 and IL-10 immunity, which allows the pregnancy to continue [11,12,13].

On the other hand, insufficient and/or inefficient remodeling of the maternal vascular bed in PE leads to placental ischemia, which promotes a chronic immune and redox imbalance, characterized by a reduced natural killer activity [14], and an increase of Th-1 response leading to an exacerbated inflammatory status [15,16]. This chronic inflammatory and hypoxic condition increase reactive oxygen species (ROS) and reactive nitrogen species (RNS) production such as O_2_^-^, OH, H_2_O_2_, NO, and ONOO^-^. These molecules are produced by cell metabolism, and under physiological conditions work as cell signaling molecules [17,18,19,20]. However, excess ROS and RNS production causes the inappropriate balance of the antioxidant defense, leading to cellular damage of proteins, lipids, and DNA, through different posttranslational modifications, such as peroxidation, S-nitrosylation, carbonylation, and protein nitration. To delve deeper into the matter of nitric oxide (NO), which is recognized as a significant endothelium-derived relaxing factor, evidence shows that the enzyme levels (eNOS) that synthesize NO are not modified in PE, yet NO bioavailability is reduced [21,22]. This observation could be explained by the transformation of NO to peroxynitrite (ONOO^-^), which is enabled by the high availability of superoxide anion (O_2_^-^) in this condition [23,24]. Therefore, the reduction in NO vascular levels decreases vascular relaxation and provides a partial explanation for the hypertensive response in PE. Moreover, molecules that increase ROS/RNS production, also decrease proliferation and invasion of the extravillous trophoblast (EVTs) [25], therefore distressing the peri-implantation period.

In PE many vasoactive factors are compromised. Thus, for example, in human omental arteries taken from women with PE, an increase in vasopressin and KCl-induced vasoconstriction and an absent acetylcholine-dependent relaxation compared with normotensive gravidas was observed [26]. Additionally, the plasma concentration of the vasoactive neuropeptide Y (NYP) was elevated in preeclamptic women compared with normal pregnancy [27]. Both vasopressin and NYP, increase Ca^2+^ concentrations in rat and human smooth muscle cells [28,29]. It is well known that intracellular Ca^2+^ plays an essential role in the endothelial-smooth muscle cell communication and vascular physiology. On the other hand, several groups have focused on how ROS/RNS and inflammatory molecules alter Ca^2+^ homeostasis and its signaling in PE [30]. In this review, we analyze how ROS/RNS could contribute to the generation of PE in the implantation period and the worsening of PE through changes in endothelial/smooth muscle cell communication mediated by connexin (Cx)-based channels.

## 3. General Characteristics of Cx-Based Channels

Cxs are a family of proteins present in almost all cell types in the human body [31]. Twenty-one different coding genes for Cxs have been described [32], which are named accordingly to their predicted molecular weight (i.e., Cx43, ~43 kDa). Despite the great diversity of the members of this family [33], they have a high amino acid sequence conservation. Cxs are formed by four transmembrane domains connected by two extracellular and one intracellular loop. Both the N- and C-terminus are located at the cytoplasmic side of the membrane [31,34]. In some Cxs, the N-terminal portion has been described inside the channel forming the intracellular pore funnel [35,36,37], where it acts as part of the voltage sensor [38].

**Figure 1:**
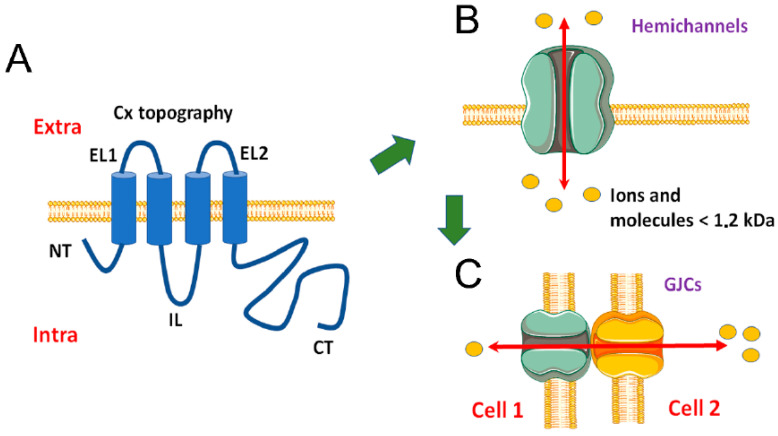
General characteristics of Cx and Cx-based channels. Panel (**A**) illustrates a representative topography of Cx at the plasma membrane. Connexin proteins are formed by four transmembrane domains, two extracellular loops (EL1 and EL2), one intracellular loop (IL), and both the N- and C-terminus are facing the cytoplasm. Panel (**B**) displays a hexamer of Cxs for a connexon or hemichannel. The channel presents a central pore through which, bidirectionally flow of ions and molecules up to ~1.2 kDa occurs. Panel (**C**) shows the formation of a gap junction channel (GJC) by serial docking of two hemichannels at the cell-to-cell contact. GJC allow the exchange of ion and molecules between the cytoplasm of neighboring cells.

Accordingly, mutations in the N-terminal modify the channel properties, such as permeability, selectivity, and open probability [39,40,41,42,43]. Concerning the C-terminal domain, it is the most variable region between Cxs, not only in their amino acid composition but also in its length. For instance, Cx23 has the shortest C-terminal, and Cx62 has the largest one among all Cxs. Additionally, the C-terminal domain is a prominent site for posttranslational modifications in Cxs [44]. Hence, most of phosphorylations [45,46,47], S-nitrosylation [48], and proteolytic cleavage sites [49,50] are found in the C-terminus. A particular feature of Cxs is that they form two types of channels, hemichannels and GJCs (Figure 1). Despite that these two types of channels can be formed by the same type of protein, they show several differences in terms of cellular localization, permeability, control mechanisms, and cell function [51].

### 3.1. Hemichannels

Hemichannels are formed by hexamers of Cxs [35,52]. These channels form a central pore by which ions and molecules up to 1.2 kDa move following their electrochemical gradient [53]. Thus, experiments performed mostly in vitro, have demonstrated that when hemichannels increase their open probability (mostly caused by a reduction in the divalent cation concentration in the extracellular media [54,55,56]), they mediate an increase in extracellular concentration of ATP [57], glutamate [58], GSH [59], lactate [60], and NAD^+^ [61], and intracellular glucose [62], Na^+^ [63], and Ca^2+^ [64,65]. Despite the initial thought that hemichannels were non-selective, it is clear now that they have selective permeability, mainly determined by the size, 3D structure, and charge of the molecule in transit [66]. For example, Cx43 hemichannels are permeable to ethidium bromide (^1+^, 394 g/mol), but are not permeable to urea or glycerol which are non-charged and smaller (60 and 92 g/mol respectively) [67]. Similarly, Cx30 hemichannels are permeable to glucose (180 g/mol) but not to water (18 g/mol) [67].

Under physiological conditions, hemichannels are mostly closed, probably to “prevent” leakage of molecules such as ATP and amino acids [68]. This low hemichannel activity is maintained mainly by extracellular Ca^2+^ and Mg^2+^ concentrations in the mM range [54,55,56] and by a negative membrane potential [53,56,69]. However, some molecules found in vivo may favor an increased but controlled hemichannel opening. Thus, hemichannels increase their activity under redox reducing conditions [70,71] as well as when stimulated by linoleic acid [72]. It is plausible that hemichannel activity in vivo is higher than reported due to a lack of appropriate conditions for determining hemichannel activity in vitro. Accordingly, there is increasing evidence that hemichannel activity in vivo is high enough to have a significant role in information processing in the central nervous system [73]. For instance, blocking Cx43 hemichannels, with a mimetic peptide, in the lateral amygdala of adult rats induced amnesia in animals under a fear conditioning protocol [74], while under normal conditions these animals would remember the distressing experience. This result was associated with a lower release of glutamate and D-serine from astrocytes [74]. Other examples of hemichannel activity under physiological conditions is the release of D-serine from astrocytic hemichannels, which regulates glutamate-induced currents in prefrontal mouse cortex neurons [75], and the release of ATP from hippocampal astrocytes through Cx43 hemichannels, which regulate the basal activity of CA1 pyramidal neurons in a mouse model [76]. These data strongly suggest that hemichannels activity is low under physiological conditions, but high enough to mediate critical cellular processes.

Under pathological conditions, hemichannel activity is notoriously increased [68,77]. Hemichannels with higher activity are known as “leaky hemichannels” [68] because they represent a leaked route for molecules from the cytosol to extracellular space through the plasma membrane. This uncontrolled leaky flux of molecules leads to cell malfunction, and in some cases, cell death [68,77]. Some mutations in Cx-genes have been associated with a massive hemichannel activity, which in turn has been linked to KID (Keratitis–Ichthyosis–Deafness) syndrome [78,79,80,81] and X-linked Charcot-Marie-Tooth disease [82]. In addition, lateralization of Cx43—forming hemichannels—contributes to cardiac arrhythmias and leads to lethality in animal models of Duchenne muscular dystrophy [77].

Our group and others have proposed that molecules associated to oxidative stress modulate hemichannel opening [70,83,84,85,86]. For example, Cx43 hemichannel S-nitrosylation exacerbates hemichannel activity in cerebral ischemia [87] and inflammatory models [62]. Despite that the molecular mechanism is unknown, Cx cysteine (Cys) oxidation has been proposed as a mediator in this phenomenon [70,86,88]. In particular, the effect of NO on Cx43 hemichannels was associated with an increase in their open probability [87], and in the case of Cx46 hemichannels, with modifications of their electrophysiological properties and changes in their permeability to large molecules [88]. On the other hand, the oxidation of a putative free Cys in one of the extracellular loops seems to induce the closure of the hemichannel by 4-HNE [86]. Interestingly, the Ca^2+^ sensor of Cx26 is located at the interface of transmembrane 1 and the extracellular loop 1 [89], and the Cx32 sensor is constituted of several asparagine residues in the extracellular loop 1 [55]. Suggesting that any posttranslational modification in the extracellular loop 1 could modulate (in any direction) the Ca^2+^ sensor effect. Recently, our group suggested that the S-nitrosylation of Cx46 hemichannels could play an important role in cataract formation [90]. In summary, pathological conditions, such as mutations and inflammation, can induce leaky hemichannels leading to cell injury.

### 3.2. GJCs

GJCs are formed by the serial docking of two hemichannels [35]. These types of channels are permeable to ions [91] and to second messenger molecules, such as Ca^2+^, IP_3_, and cAMP [92,93,94,95,96], glucose [97,98], small peptides [99,100] and miRNAs [101,102]. Under physiological conditions, GJCs allow metabolic and electrical coordination between neighboring cells, which are essential for correct tissue functioning. Proinflammatory conditions have been associated with a decrease in gap junctional communication between cells in different tissues, such as brain, lung, and liver [62,103,104,105]. In contrast, other pathological conditions such as brain ischemia, where gap junctional coupling is maintained, this route of communication serves as a way to spread damaging signals to healthy cells [106] in a sort of “bystander effect”. Accordingly, evidence shows that astroglial Cx43 GJCs mediate neuronal and glial death in regions far beyond the infarcted zone [106,107,108]. Therefore, maintaining cellular communication is critical for retaining normal tissue function. However, under pathological conditions, the characteristics of gap junction-mediated communication could enhance or diminish cell injury to neighboring cells, probably depending on the nature of the injury. The effect of free radicals on GJC communication is not clear, but in general, is associated with a decrease of intercellular coupling [62,109,110].

## 4. The Role of Hemichannels and GJCs in the Vasculature

Human and animal studies have evidenced that Cx37, Cx40, Cx43, and Cx45 are expressed in the vascular system, and play an important role in its physiology [111,112]. Despite some differences in localization due to vessel size, vascular territory, and species [111,113], it is accepted that Cx37 is mostly confined to endothelial cells and is expressed to a lesser degree in smooth muscle cells [114,115]. Cx40 is expressed in endothelial and smooth muscle cells [116,117], Cx43 is relatively evenly distributed in endothelial and smooth muscle cells [97,100], and Cx45 is only present in smooth muscle cells [118]. The coupling between smooth muscles via GJCs [119] allows not only metabolic but also electrical coordination between cells, which in turn helps to synchronize the muscle contraction to obtain a meaningful response [120]. Also, smooth muscle cells are coupled to endothelial cells [121,122,123], producing a cellular syncytium in which cellular interactions converge in the control of the vascular tone [124]. The role exerted by Cxs has been evaluated with Gap27, a mimetic peptide that blocks Cx37 and Cx43 channels. When applied to the ex vivo system of a mesenteric resistance artery, a decrease in basal myogenic vasoconstriction was observed [124], suggesting that a “signal” is transferred from endothelial cells to smooth muscle cells via GJCs [121]. As mentioned before, GJCs are permeable to ions and signaling molecules; therefore, there are several candidates to be “the signal” crossing from endothelial to smooth muscle cells, with the potential to control the myoendothelial tone. One of the most important signaling molecules which participates in the vascular tone is Ca^+2^, which initiates the contraction in smooth muscle. The elevation of Ca^+2^ concentrations within the endothelium activates a negative feedback loop by increasing the synthesis of vasoactive molecules such as NO and endothelium-derived hyperpolarization factor (EDHF), promoting the relaxation of the vessels [125]. EDHF, an endothelium-derived vasodilator, is crucial in small arteries, and although there is no clarity as to the precise nature of this molecule, it seems that it is permeable through gap junctions, since a good correlation between the presence of functional gap junction and the activity of EDHF [126,127,128] has been demonstrated. Another possible “signal” moving through GJCs is IP3. Indeed, GJCs formed y Cx43 are permeable to IP3 [48]. This allows IP3 to flow from smooth muscle to endothelial cells, where it increases intracellular Ca^2+^ concentration. In turn, this change in intracellular Ca^2+^ activates NO production [129] which travels back to smooth muscle cells inducing vasorelaxation [48,130]. There is a dogma that claims, that the NO produced by endothelial cells “crosses freely” the plasma membranes until it reaches the cytoplasm of smooth muscle cells [131]. However, Figueroa et al. [132] suggested that NO uses Cxs 37, 40, and 43-hemichannels to easily permeate the plasma membrane of endothelial and smooth muscle cells, playing a pivotal role in the NO-dependent control of vascular tone. Furthermore, at the myoendothelial junction, NO autoregulates its transit, modifying cysteine residues on GJ channels to alter its permeability, through posttranslational modifications such as S-nitrosylation or denitrosylation, which makes channels more and less permeable, respectively [48]. It should be noted that Cxs not only facilitate the movement of NO through membranes but in turn, they can affect NO production. For instance, Cx37 interacts with eNOS, decreasing its activity and therefore decreasing NO levels [133]. Additionally, Cx40^-/-^ mice have lower levels of eNOS compared to wild type mice [134]. In summary, GJCs and hemichannels formed by Cxs, communicate and coordinate chemical and electrical signals involved in the contraction and relaxation of the vessels through protein–protein interactions and facilitating the diffusion of vasoactive molecules.

## 5. Role of Cx-Based Channels in the Placenta

The placenta is an organ that allows the exchange of metabolites, gases, and waste products between the fetus and the mother. Cx32, Cx37, Cx40, Cx43, and Cx45 are expressed in human placenta [135,136,137,138,139,140,141,142,143] (Table 1). While Cx32 and Cx43 were described in the syncytiotrophoblast, a multinucleated cell in the placental bed, yet no function has been attributed to these Cxs in this particular location [141]. On the other hand, Cx43 has been described mostly forming GJCs between cytotrophoblast cells and between cytotrophoblast cells and syncytiotrophoblast [135], where it seems to regulate the fusion of cytotrophoblast into syncytiotrophoblast [142], warranting placental growth and maturation [143]. Two possible mechanisms of action have been proposed in this process. Firstly, there might be a “signal(s)” that moves between cells that allows cell fusion and, therefore, syncytiotrophoblast formation. Secondly, Cx43 coordinates the effect of other proteins such as ZO-1 through protein–protein interactions [138]. Lastly, these two mechanisms may act together in a concerted manner to achieve cytotrophoblast fusion and syncyalization.

Cx37 and Cx40 have been described in endothelial cells derived from placental vessels [137]. Although there is no evidence of their role in this particular territory, it is likely that Cx37 and Cx40 do not deviate far from the function established in other vascular regions, i.e., working as key molecules in controlling vascular tone. Cx40 and Cx45 are prominently expressed in the EVT of anchoring cell columns [136]. In these cells, Cx40 supports EVT proliferation and prevents their differentiation into an invasive phenotype. Meanwhile, Cx45 expression is restricted to placentas before nine weeks of gestation, but its role is unknown [136,143,144].

## 6. Could Hemichannels and GJCs Mediate Part of the ROS/RNS Response in Preeclampsia?

**Figure 2:**
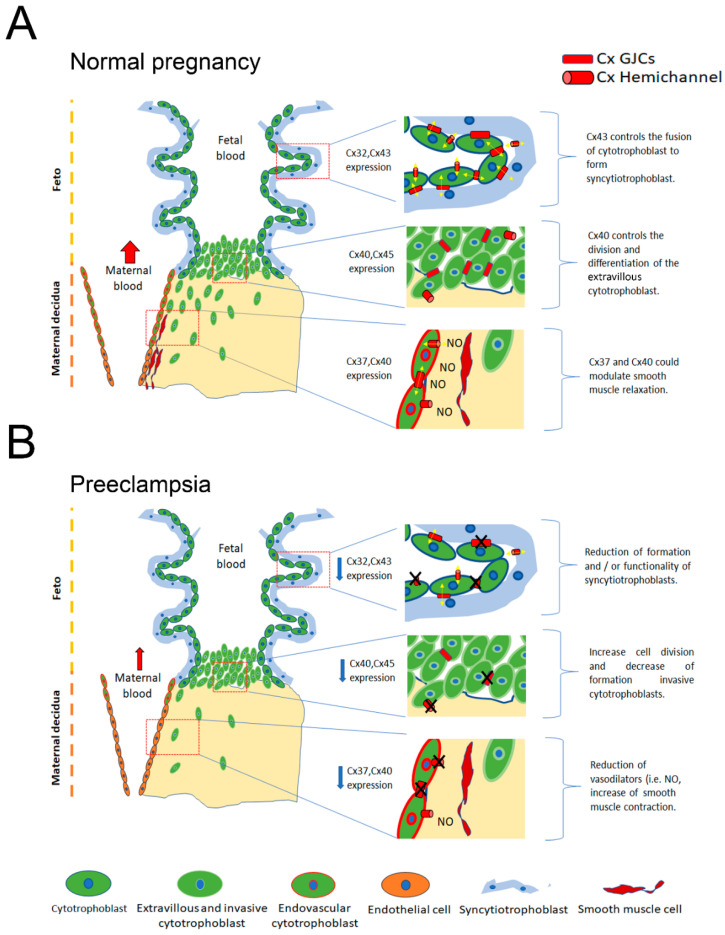
(**A**) In a normal pregnancy, Cx43 via GJCs y/o hemichannels controls the fusion of cytotrophoblast, to form syncytiotrophoblast. Cx40 controls the rate of cell division and differentiation of extravillous cytotrophoblast. Cx37 and Cx40 present in the endovascular cytotrophoblast could modulate smooth muscle relaxation via releasing vasoactive molecules such as NO. (**B**) in PE, the reduction in the expression and/or functionality (denoted by a black X) of the Cx43 GJC and hemichannels mediated by posttranslational modifications induced by ROS/RNS. Potentially, it could alter the formation and/or functionality of syncytiotrophoblast. Alterations of Cx40-based channels could reduce invasive cytotrophoblast cells, and finally, the reduction in expression/functionality of Cx37 and Cx40-based channels in the vasculature could increase smooth muscle basal contraction.

We analyze here the effect of inflammatory mediators (ROS/RNS) in Cxs-based channels function and how these modifications might be associated with vascular alterations in preeclamptic placentas (Figure 2).

**Cx37:** Even though Cx37 has been found in endothelial cells of chorionic arteries, its function has not been characterized in this particular territory. Furthermore, the presence and/or expression of this protein has not been evaluated in the placenta of preeclamptic patients.

In the presence of NO, Cx37-mediated gap junction communication is diminished [125,145,146]. Pogoda and co-workers demonstrated that in HeLa cells, this mechanism includes the inhibition of a tyrosine phosphatase (SHP-2), which removes the phosphorylation in tyrosine residue 332 on the C-terminal region of Cx37. This reduction in SHP-2 activity associates with a decrease in intracellular Ca^2+^ exchange between endothelial and smooth muscle cells [125]. The latter may represent a reduction of intercellular communication with other molecules as well.

Since NO bioavailability in PE is decreased, it could be suggested that the intercellular communication mediated by Cx37 could be increased. If the whole issue was centered on NO, it would be very counter-intuitive for the PE setting, because it would mean that the pathway for NO and other vasodilator molecules would be increased. However, as previously stated, there is no evidence demonstrating the presence and/or variation in expression of Cx37 in PE placentas nor the effect of other ROS/RNS on the activity of Cx37 GJCs. Therefore, in PE, in addition to posttranslational modifications in this Cx, a reduction of its expression could be suggested.

Contrary to the effect of NO on GJCss, in rat endothelial cells, NO increases Cx37 hemichannels activity and is permeable through them [132], suggesting a sort of paracrine signaling pathway. In preeclampsia, where NO levels are decreased, Cx37 hemichannel activity would be reduced, therefore diminishing one of NO “paracrine” releasing pathways. This mechanism is supported by evidence in a mouse Cx37-KO model, where a reduction of NO release was observed when compared with wild type animals [147].

**Cx40**: As previously mentioned, Cx40 may be found in two key locations in the placenta. First, being a part of EVT anchoring columns, where it colocalizes with proliferation markers, such as ki67. In this site, the presence of Cx40 seems to prevent the invasive pathway of EVT. As far as we know, there are no reports showing an effect of redox changes upon Cx40 levels or activity in EVT in preeclampsia. However, in rat brain tissue, oxidative stress increases Cx40 protein levels, while maintaining the same amount of mRNA, and that prevention of oxidative stress with N-acetylcysteine inhibits the increase in Cx40 protein seen without treatment [148,149]. Hence, oxidative stress in PE may be the cause of posttranslational modifications and/or an increase of protein expression of Cx40, which prevents the invasive pathway of EVT.

Second, Cx40 is found in placental and maternal vasculature. It has been observed that the lack of Cx40 (Cx40^-/-^ mice) decreases NO production in a renin-dependent hypertension model [150]. This finding suggests that Cx40 may play a role in NO production and that the lack of Cx40 may exacerbate the hypertension response through the downregulation of vasodilator molecules. However, in another study, lack of Cx40 was associated with angiotensin II independent hypertension, and it did not correlate to NO or another endothelial-derived vasodilator [151]. This suggests that there might be a non-canonical pathway by which Cx40 is associated with hypertension without interfering with vasodilator molecules passage between neighboring cells. Also, a NO/cGMP-dependent de novo formation of Cx40-mediated gap junction coupling was observed in HeLa and HUVEC cells [152,153]. Based on these results, we could suggest that the reduction of NO levels seen in preeclampsia could impact the Cx40-mediated gap junction by reducing its coupling. This might not only exacerbate the reduced levels of NO by interfering with its propagation in the muscle-endothelial junction but also exacerbate the hypertensive response by interfering with the action of other vasodilator molecules such as EDHF. Regarding hemichannels, in rat endothelial cells, NO increases Cx40-hemichannel activity and permeates through them [132]. Thus, a decrease of NO bioavailability in PE could decrease Cx40-hemichannel activity, while also reducing the release of NO and its mediated vasodilatation, exacerbating once again the hypertensive response.

**Cx43**: In pregnant sheep, increased Cx43 gap junction function was associated with an increase in sustained Ca^2+^ burst and NO-induced vasodilatation [154]. In this work, Gap27 (which has been used as a hemichannel and GJC blocker) was used as a specific inhibitor of Cx43 [155,156]. Even though the authors claim that the effect was mediated by GJCs [154], the participation of hemichannels cannot be discarded. Moreover, the opening of Cx43 hemichannels mediates the uptake of Ca^2+^ from the extracellular space [64]. Taking into account these possibilities, we suggest that in normal pregnancy, Cx43-hemichannel and/or GJC functions, increase intracellular Ca^2+^ dynamics, which in turn increases NO production and its vasodilatory effect. Therefore, the exacerbated prooxidant and inflammatory milieu in PE could induce posttranslational modifications in Cx43, decreasing (in permeability or open probability) the hemichannel and/or GJCs function, reducing the vasodilator capacity of endothelial cells. Additionally, proinflammatory molecules can decrease Cx43 expression [157,158], therefore in PE, a similar phenomenon could be expected.

On the other hand, NO can increase Cx43 hemichannel activity in astrocytes. If the same mechanism operates in PE placentas, a decrease in Cx43 hemichannel activity in the syncytiotrophoblast layer of the placenta, due to lower bioavailability of NO, could be expected. Additionally, since NO uses Cx43-hemichannels as a pathway between the intra and extracellular space, a decrease in the release of NO could be expected as well [132]. As mentioned before the IP3 permeability through Cx43 GJCs is modulated by the S-nitrosylation of C271 [48]: Therefore, a decrease in the bioavailability of NO in PE could maintain Cx43 GJCs mainly in a closed state [48]. This could affect, at least theoretically, the flow of IP3 between smooth muscle and endothelial cells and also reduce the diffusion of EDHF and other vasodilator molecules, and as a consequence an increase in the placental vascular resistance.

Lower Ca^2+^ and Mg^2+^ plasma levels correlate with a higher risk of developing PE [159,160]. Accordingly, Ca^2+^ supplementation might decrease the risk of hypertension and therefore PE [161,162]. The hemichannel opening is controlled by intracellular and extracellular Ca^2+^ and Mg2+ concentrations, where a decrease of extracellular divalent cations increases hemichannel opening [55,163]. In consequence, a reduction of the extracellular concentration of these two ions in PE, could increase hemichannel opening. On the other hand, Cx43 hemichannels are permeable to ATP [164], and in a condition with an increased hemichannel open probability, a higher extracellular ATP concentration could be expected. This extracellular nucleotide, activates P2X and some P2Y receptors, and both can increase intracellular Ca^2+^ concentration [165]. In a healthy pregnancy, extracellular ATP induces intracellular Ca^2+^ bursts, modulating NO production [166]. However, in human preeclamptic umbilical endothelial cells, the Ca^2+^ and NO response to extracellular ATP is much lower [167]. A possible explanation of this, is that a sustained efflux of ATP through hemichannels, could induce P2 receptors desensitization, and therefore a loss of Ca^2+^ response and NO production.

Additionally, Cx43 GJCs seems to potentiate the role of Ca^2+^ as an NO inductor, via coordination of endothelial cell responses [154]. Usually, in experiments that mimic inflammatory and/or oxidative conditions, Cx43 GJCs come close together [62,109,110]. Therefore, in PE inflammatory and/or oxidative conditions could decrease Ca^2+^-induced NO production, via inhibition of GJCs communication.

As mentioned before, GJCs are permeable to second messenger and molecules such as miRNAs. There are several miRNAs that are associated with trophoblastic differentiation, migration and invasion [168]. Cx43 GJCs are permeable to several miRNAs [102,169]. Interestingly, Cx43 GJCs allows the exchange of miRNAs between bone-marrow-derived mesenchymal stem cells or cancer cells and endothelial cells [101,170,171]. Therefore, if Cx43 GJCs communication is reduced in PE (either because they are closed or their expression is reduced), a decrease of the flux of these molecules could be expected and malfunctioning of trophoblast’s function.

**Cx32 and Cx45**: There is no information regarding the role of Cx32 and Cx45 in preeclampsia. Moreover, the information about how ROS and RNS molecules affect the activity of these proteins in the placental vasculature is scarce. The need for experiments that would shed light on the role of these channel proteins in the placental vascular tone in PE is evident.

## 7. Conclusions and Future Directions

There is extensive evidence demonstrating that Cxs play a pivotal role in the regulation of systemic vascular function. Although the information regarding the role and function of Cxs in the placental vascular bed is scarce, this does not mean that Cxs are less relevant in this organ. On the contrary, we propose that Cxs might be directly involved in the maternal hypertensive disorder in response to an insufficient trophoblast invasion in PE.

**Figure 3:**
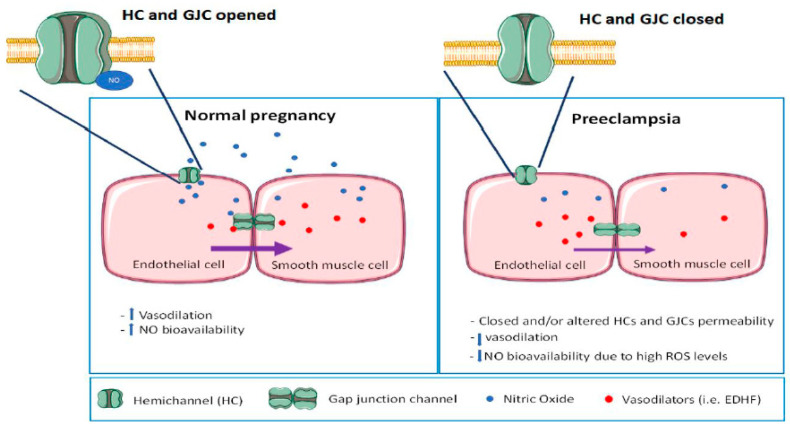
Reduction of Cx-based channel activity in preeclampsia. Normal pregnancy displays physiological NO bioavailability, with physiological hemichannels (HC) and gap junction channels (GJCs) activity that supports the exchange of NO, endothelium-derived hyperpolarization factor (EDHF), and other molecules between endothelial and smooth muscle cells. Consequently, blood flow to the placenta is adequate to satisfy its metabolic needs. However, NO bioavailability is reduced in preeclampsia due to increased free radicals. Hemichannels (HC) and gap junction channels (GJC) decrease their open probability and/or expression, reducing the flux of vasodilator molecules between endothelial and smooth muscle cells.

We have analyzed the effect of oxidative stress, a key feature of preeclampsia, on Cxs in other territories. It is plausible that Cxs posttranslational modifications induced by ROS/RNS might be key elements in the vascular pathophysiology in PE. Thus, we suggest that the overall effect of ROS/RNS in the placental vascular cells is to decrease Cx-formed channels activity, which in turn reduces NO bioavailability and vasodilatation (Figure 3). In addition, it is possible that ROS/RNS cause lateralization of Cxs so that Cxs may be found in an aberrant location, i.e., increasing the expression of hemichannels while reducing gap junction Cxs—as it occurs in cardiac muscle Cx43 of Duchenne muscular dystrophy mice [77]. The possible increase in mislocalized hemichannels would increase the loss of NO to extracellular space, and reduce NO vasodilatory capacity. This review included results obtained in animals as well as humans. Research focused on the role of Cxs in women with PE is scarce; therefore, future investigations regarding the molecular mechanisms that modulate the Cx function in PE are needed. This review is an attempt to provoke the study not only of the effect of Cx dysregulation in the human placenta, but also to investigate the factors that induce these changes. Additionally, we advocate for the evaluation of non-canonical Cx functions and channel properties, as there could be a direct role of these proteins in trophoblast invasion and proliferation. Consequently, we encourage developing and enhancing new research on the C-terminal tail of Cxs, i.e., the target of this type of protein modifications in the preeclamptic placenta.

## Figures and Tables

**Table 1:** Expression and role of Cxs in human placenta. The localization and attributed functions of the Cxs expressed in placenta are listed. The assay applied for detection is also listed.

Cx Type	Localization	Assay	Attributed Function	Reference
**Cx32**	Placenta tissue	PCR	unknown	[135]
	cytotrophoblastic culture	Inmmunolocalization		[136]
				[141]
**Cx37**	Endothelial cells from	PCR	unknown	[136]
	chorionic arteries			
**Cx40**	Endothelial cells from	PCR	Cytotrophoblast proliferation	[135]
	placenta arterioles	In situ Hybridization		[136]
		Inmmunolocalization		[137]
	EVT cells in all	Northern blot	Epithelial like	
	anchoring columns		trophoblast marker	
**Cx43**	Placenta tissue	PCR	Fusion of cytotrophoblast to	[136]
		Northern blot	form syncytiotrophoblast	[137]
		In situ Hybridization		[139]
		Inmmunolocalization		[141]
				[142]
**Cx45**	Placenta tissue	PCR	unknown	[136]
		Inmmunolocalization		[143]
				[144]
